# Case Report: Community-Acquired *Legionella gormanii* Pneumonia in an Immunocompetent Patient Detected by Metagenomic Next-Generation Sequencing

**DOI:** 10.3389/fmed.2022.819425

**Published:** 2022-01-28

**Authors:** Cheng Lei, Xianglin Zhou, Shuizi Ding, Yingjie Xu, Binyi Yang, Wei Guo, Min Song, Min Yang, Yunan Jia, Hong Luo

**Affiliations:** ^1^Department of Pulmonary and Critical Care Medicine, The Second Xiangya Hospital, Central South University, Changsha, China; ^2^Research Unit of Respiratory Disease, Central South University, Changsha, China; ^3^Hunan Diagnosis and Treatment Center of Respiratory Disease, Changsha, China

**Keywords:** metagenomic next-generation sequencing (mNGS), *Legionella gormanii*, community-acquired pneumonia, pathogen diagnosis, bronchoalveolar lavage fluid

## Abstract

**Background:**

*Legionella* spp. has been well-recognized as an important cause of community-acquired pneumonia. Current community-acquired pneumonia guidelines recommended covering the treatment of *Legionella* because of the high mortality associated with inadequate antibiotic treatments. However, the symptom of *Legionella* pneumonia is non-specific, and routine diagnostic tests exhibit low sensitivity for *Legionella* spp., especially for non-*Legionella pneumophila* serogroup 1 strains.

**Case Presentation:**

We report a 53-year-old man without underlying diseases admitted to respiratory intensive care unit because of severe community-acquired pneumonia and respiratory failure. Although, the results of routine culture of bronchoalveolar lavage fluid and the *Legionella* urinary antigen test were all negative, metagenomic next-generation sequencing (mNGS) identified a great amount of DNA and RNA sequences of *Legionella gormanii* in bronchoalveolar lavage fluid while negative in blood sample. The presence of *Legionella gormanii* in bronchoalveolar lavage fluid was further confirmed by polymerase-chain-reaction and Sanger sequencing.

**Conclusion:**

*Legionella gormanii* has rarely been reported in patients with community-acquired pneumonia mainly due to lack of diagnostic test for non-*Legionella* pneumophila serogroup 1 strains. This is the first report of *Legionella gormanii* pneumonia in an immunocompetent patient detected by mNGS, which indicates that mNGS is a high-resolution and sensitive assay for the diagnosis and surveillance of *Legionella* infection.

## Introduction

*Legionella* is a Gram-negative fastidious bacterium and a common cause of atypical community-acquired pneumonia (1). The mortality rate of community-acquired *Legionella* pneumonia has decreased from 26% in 1985 to 10% in 1998, which may relate to the development of the sensitive urine antigen test or the changes in empirical antibiotic treatment strategies (2). However, the genus *Legionella* comprises more than 65 different species (3), and the current widely used urine antigen test is only available to detect *Legionella pneumophila* serogroup 1 (4). The diagnosis of other serogroups and non-*Legionella* pneumophila species infection is difficult.

Metagenomic next-generation sequencing (mNGS) can directly identify the pathogens' sequences in clinical samples and has been applied in clinical infectious disease diagnostics including pneumonia (5–7). Here, we report a case of severe community-acquired pneumonia in a 53-year-old man, in which the conventional pathogen-detecting tests were all negative including legionella urinary antigen test, while a great amount of DNA (7,957 in a total of 21,977,576 sequences) and RNA (3,443 in a total of 12,763,560 sequences) sequences of *Legionella gormanii* in bronchoalveolar lavage fluid were identified by mNGS. After quinolones antibiotic therapy, the patient recovered.

Only five cases of *Legionella gormanii* pneumonia have been reported in PubMed, and all these reported patients were immunosuppressed (8–12). The present case represents the first case of community-acquired *Legionella gormanii* pneumonia in an immunocompetent patient detected by mNGS.

## Case Description

**Figure 1:**
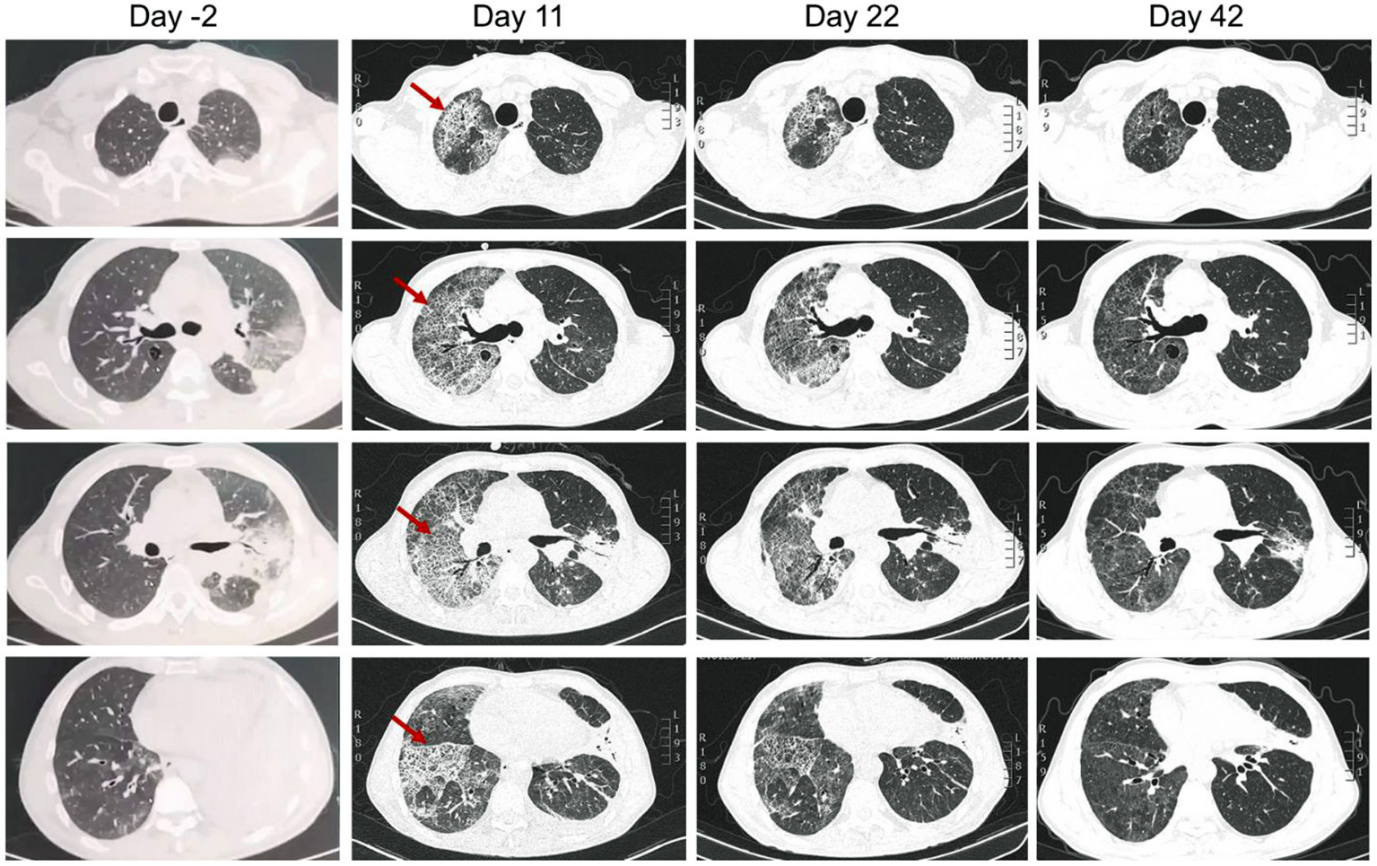
Chest computed tomography of the patient on 2 days before admission (day −2), and day 11, 22, and 42 (20 days after discharge) after admitted to our hospital. Arrows indicated the right lung developed interstitial infiltrates on day 11.

A previously healthy, 53-year-old male patient presented with a 7-day history of cough and chest pain, then a 4-day history of fever and dyspnea. He was referred to respiratory intensive care unit on September 28, 2021 (day 1). The patient had a smoking history of 40 years with a pack of cigarettes every day. He was a handyman but denied recent travel history, or any cooling systems or other special man-made water system exposure. A chest computed tomography (CT) taken on September 26 (day −2) showed consolidation in the left upper lobe and left lower lobe, left pleural effusion, and bullae in the posterior segment of the right upper lobe (Figure 1). Since he presented with hypoxia, high-flow nasal cannula oxygen therapy was used.

**Figure 2:**
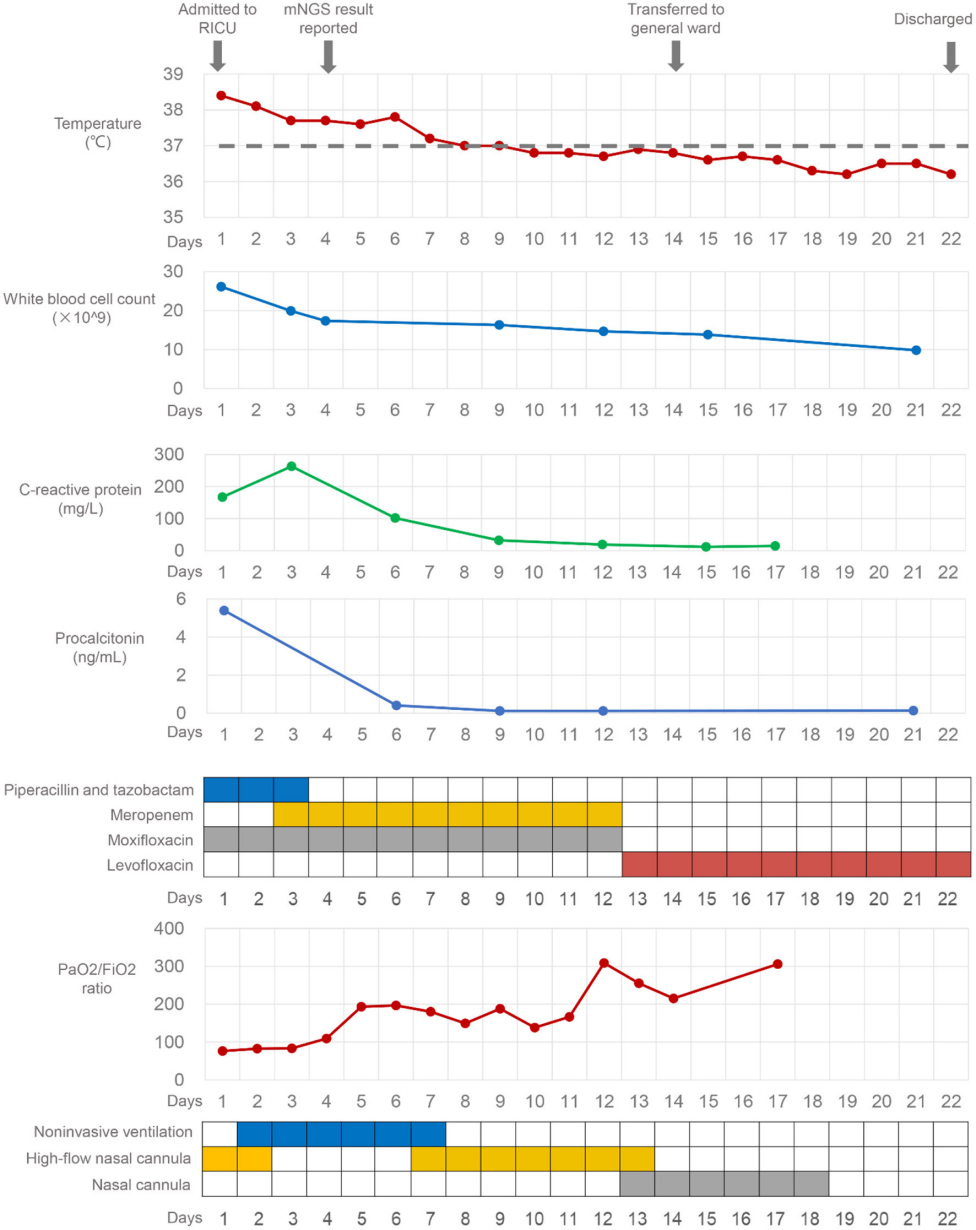
The clinical course of the patient with *Legionella gormanii* infection.

On admission, the laboratory examination results were as follows: white blood cell (WBC) count was 26.12 × 10^9^/L, with a neutrophil percentage of 93.6%, the concentration of procalcitonin (PCT) was 5.39 ng/mL, the level of C-reactive protein was 167.21 mg/L, the CD4+ lymphocyte count was 245/μl, and the serum β-(1,3)-D-glucan and galactomannan were normal. The empiric antibiotic therapy included piperacillin-tazobactam and moxifloxacin (Figure 2). On day 2, his hypoxia progressed and he started to be supported by noninvasive ventilation with 90% FiO_2_, inspiratory positive airway pressure of 12 cm H_2_O, and expiratory positive airway pressure of 8 cm H_2_O. To search the infectious etiology of pneumonia, bronchoalveolar lavage fluid (BALF) were harvested and sent with a blood sample to perform mNGS (KingMed Diagnostics, Changsha, China). The methods of BALF and blood mNGS were described in Supplementary File. The BALF culture for bacteria and fungi, galactomannan, polymerase-chain-reaction (PCR) for *Mycobacterium tuberculosis*, and *Legionella* urine antigen test (KingMed Diagnostics, Guangzhou, China) were negative. On day 3, the antibiotics were adjusted to meropenem and moxifloxacin, which were more broad-spectrum.

**Figure 3:**
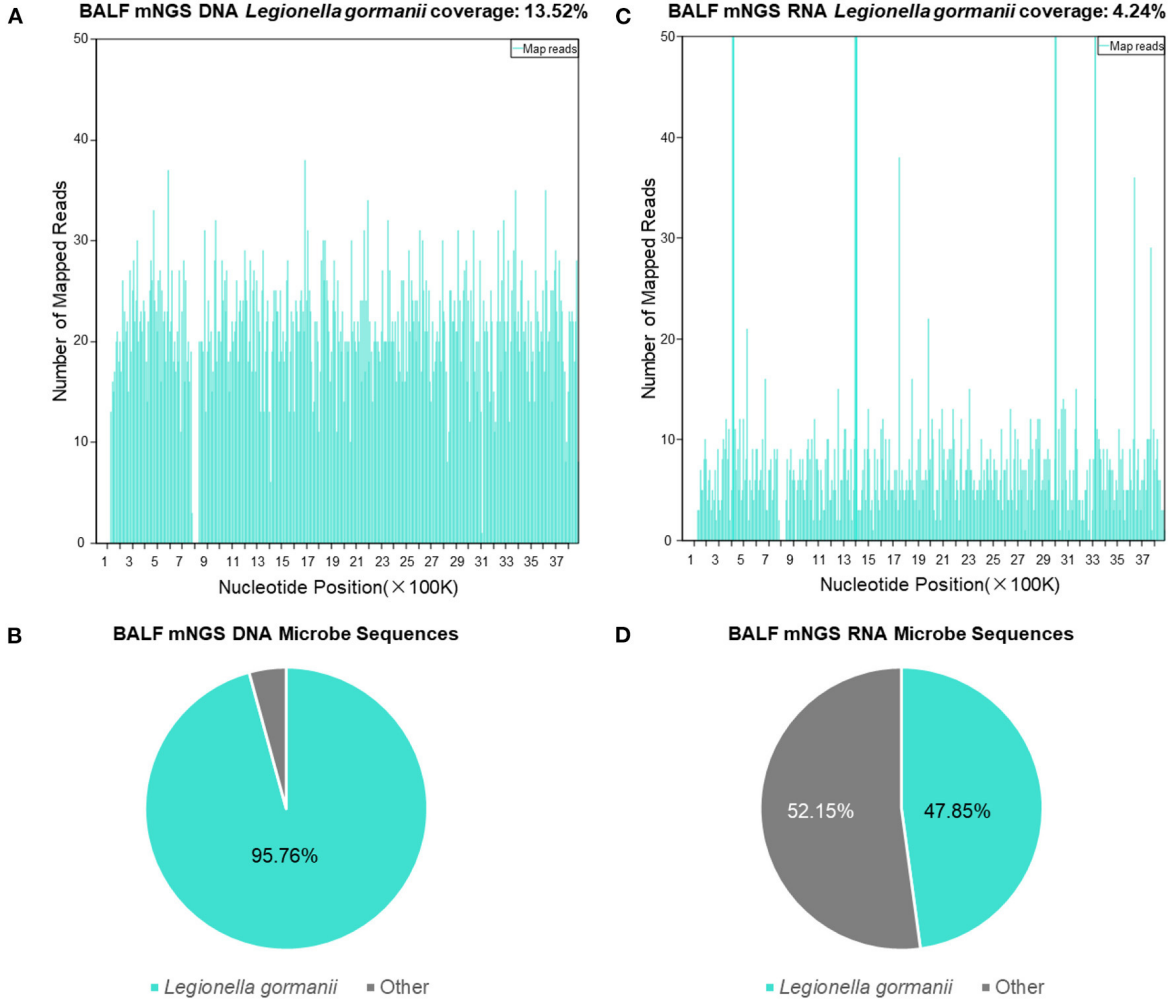
The bronchoalveolar lavage fluid mNGS results of the patient. **(A)** The DNA mNGS results showed that the coverage of *Legionella gormanii* was 13.52%. **(B)** 95.76% of the microbe DNA sequences were *Legionella gormanii*. **(C)** The RNA mNGS results showed that the coverage of *Legionella gormanii* was 4.24%. **(D)** 47.85% of the microbe RNA sequences were *Legionella gormanii*.

**Figure 4:**
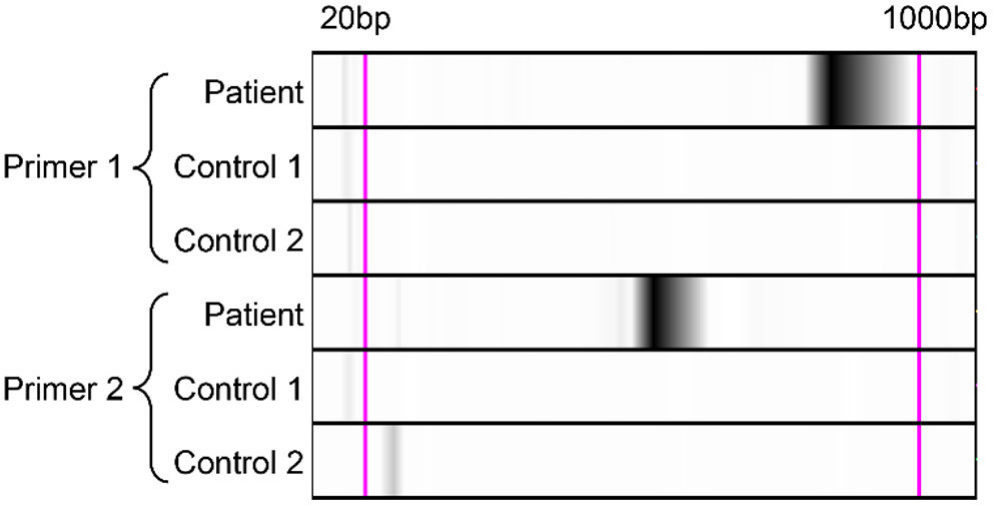
Polymerase-chain-reaction and the capillary electrophoresis technique confirmed the *Legionella gormanii* infection in the patient. Lane Patient: the bronchoalveolar lavage fluid sample of the patient; Lane Control 1: the blank control; Lane Control 2: the negative control, the bronchoalveolar lavage fluid sample from a patient who was finally diagnosed without *Legionella* pneumonia.

The BALF mNGS results reported *Legionella gormanii* infection on day 4 (Figure 3). DNA mNGS detected 7,957 sequences which can be mapped to *Legionella gormanii* in a total of 21,977,576 sequences, and the coverage was 13.52%, making up 95.76% of the total microbe sequences (Figures 3A,B). RNA mNGS detected 3,443 sequences which can be mapped to *Legionella gormanii* in a total of 12,763,560 sequences, and the coverage was 4.24%, making up 47.85% of the total microbe sequences (Figures 3C,D). However, the blood mNGS results for DNA and RNA were both negative. Targeted PCR of *Legionella gormanii* in BALF using two pairs of primers was applied: Primer1 forward 5′-CTCCGCCCACATCAATCGTA-3′, reverse 5′-AAACCCGAACTCATGGCAGT-3′; Primer2 forward 5′-GAAAGTGGTATGGTGCGGGA-3′, reverse 5′-CTCTTCACGACTTGCACCCT-3′. The primers were designed and verified using Primer-BLAST (https://www.ncbi.nlm.nih.gov/tools/primer-blast/) based on the reference genome sequence of *Legionella gormanii* in NCBI (https://www.ncbi.nlm.nih.gov/). The capillary electrophoresis technique (Qsep 100^TM^; Bioptic) and Sanger sequencing confirmed the *Legionella gormanii* infection (Figure 4). The patient was finally diagnosed with community-acquired *Legionella gormanii* pneumonia.

After 1 week of therapy, the patient's body temperature, hypoxia, and inflammatory biomarkers improved (Figure 2). Chest CT on day 11 showed consolidation in the left upper and lower lobe of the lung improved, while the right lung developed interstitial infiltrates (Figure 1). On day 13, the oxygen therapy was changed to nasal cannula, and the antibiotics were de-escalated to levofloxacin monotherapy due to mild elevated liver enzyme (ALT 175.7 U/L, AST 82.8 U/L). The patient was transferred to the general ward on day 14. Chest CT on day 22 showed the infection had improved, and subsequently, the patient was discharged. He took oral levofloxacin therapy at home for 3 weeks, and the chest CT on day 42 (20 days after discharge) showed marked improvement in the lung (Figure 1). The patient recovered well without any complications, and he had return to work and normal activities on the last follow-up (2 months after discharge).

## Discussion and Conclusions

*Legionella* spp. is an important etiology of community-acquired pneumonia, and *Legionella pneumophila* causes ~90% of all reported cases, 79% of which are caused by *Legionella pneumophila* serogroup 1 (13). Non-*Legionella pneumophila* spp. infection occurred more commonly in patients with immunosuppression than immunocompetent patients, in which the most common species are *Legionella micdadei, Legionella bozemanae*, and *Legionella dumoffii* (14). *Legionella gormanii*, previously known as *Fluoribacter gormanii*, was the first non-*Legionella pneumophila* species isolated in China (15), however, it has not been reported previously in Chinese patients, and this case is the first to report *Legionella gormanii* pneumonia in an immunocompetent patient.

Culture is the gold standard to diagnose *Legionella* infection, but only experienced laboratories can isolate *Legionella* successfully, and its sensitivity is highly variable ranging from <10 to 80% (4). Legionella urinary antigen test is the most widely used method to diagnose *Legionella* infection, and it has largely improved the diagnostic rate of *Legionella* infection. The sensitivity of the urinary antigen test ranged from 70 to 100%, and its specificity can approach 100% (16). However, the urinary antigen test is only reliable for the detection of *Legionella pneumophila* serogroup 1. Metagenomic next-generation sequencing may circumvent the disadvantages of culture and legionella urinary antigen test as it can profile the pathogens comprehensively. mNGS achieves higher taxonomy resolution compared to 16S rRNA gene sequencing and has been applied to pneumonia diagnosis, outbreak tracking, infection control surveillance, and pathogen discovery (5). The clinical manifestations of *Legionella pneumonia* are non-specific, and the diagnosis mainly relies on the laboratory pathogen examination. In our patient, the urinary antigen test was negative, and our laboratory was not able to conduct the *Legionella* culture. *Legionella gormanii* was detected by DNA and RNA BALF mNGS, and subsequently targeted PCR and Sanger sequencing validated *Legionella gormanii* infection. Although the current cost of mNGS (about 3500 Ren Min Bi per sample in China) is higher compared with any single traditional microbiological test, the benefit of its short turn-round time and the precise pathogenic diagnosis may have the potential to reduce the overall hospital costs through optimal patient management.

Only five previous case reports were identified in PubMed describing patients with *Legionella gormanii* pneumonia (Table 1). All these patients were immunosuppressed and had underlying diseases. Three of these patients were diagnosed by BALF culture, one by direct fluorescent antibody staining of the pleural exudate, and one by Fluorescent *in situ* Hybridization of the lung biopsy specimen. Although, the role of rifampin combination therapy was inconclusive and may increase adverse events (17), four of these patients used rifampin-based combination therapy, one of whom died. Our patient was the first immunocompetent patient diagnosed with *Legionella gormanii* pneumonia through mNGS and successfully recovered after fluoroquinolones monotherapy.

**Table 1:** Summary of cases with *Legionella gormanii* pneumonia.

**Publication year**	**Gender**	**Age**	**Immunosuppressed status**	**Underlying diseases**	**Radiological features**	**Diagnostic test**	**Targeted antimicrobial therapy**	**Prognosis**
1988 (8)	Female	64	High-dosage prednisone therapy	Systemic lupus erythematosus, adenocarcinoma	Right lower lobe pneumonia	Culture of bronchoalveolar lavage fluid	Erythromycin	Recovered
1989 (9)	Male	6	Primary immunodeficiency	Chronic granulomatous disease	Consolidation in the left lower and left upper lobes and a small left pleural effusion	Direct fluorescent antibody staining of the pleural exudate	Erythromycin and rifampin	Recovered
1994 (10)	Male	47	Oral prednisone (10 mg every other day), chronic lymphocytic leukemia	Chronic lymphocytic leukemia	Right middle and lower lobe infiltrate	Culture of bronchoalveolar lavage fluid	Erythromycin and rifampin	Recovered
2004 (11)	Male	75	Treatment with corticosteroids and methotrexate	Diabetes, dermatosis	Infiltrate in the right upper lobe, and subsequently developed bilateral basal infiltrates	Fluorescent in Situ Hybridization of the lung biopsy specimen	Erythromycin and rifampin	Died
2006 (12)	Male	54	Chronic lymphocytic leukemia	Chronic lymphocytic leukemia	Infiltration with nodular opacity at the left lower lobe	Culture of bronchoalveolar lavage fluid	Ofloxacine and rifampin	Recovered
This study	Male	53	No	No	Consolidation in the left upper and lower lobes and left pleural effusion, and subsequently developed infiltrates in both lungs	mNGS of the bronchoalveolar lavage fluid	Moxifloxacin, levofloxacin	Recovered

Multiple outbreaks of *Legionella* infections have been reported previously, but person-to-person spread was only reported in one case (18). Contaminated water or contaminated soil can be a source to transmit *Legionella* infection (19), however, the sources of infection remained unknown in all previously reported *Legionella gormanii* pneumonia. As a rapid and unbiased approach to detect microorganisms, mNGS also have the potential to identify pathogens directly from the environment. Further studies are warranted to explore the sources of *Legionella gormanii* infection using a metagenomic approach.

In summary, our study presented an immunocompetent patient with severe *Legionella gormanii* pneumonia diagnosed by mNGS while the *Legionella* urinary antigen test was negative, and the patient recovered after fluoroquinolones monotherapy. mNGS may be a high-resolution and sensitive assay for the diagnosis and surveillance of *Legionella* infection.

## Ethics Statement

The studies involving human participants were reviewed and approved by Review Board of the Second Xiangya Hospital of Central South University in China. The patients/participants provided their written informed consent to participate in this study. Written informed consent was obtained from the individual(s) for the publication of any potentially identifiable images or data included in this article.

## Author Contributions

CL, XZ, and SD collected the data and wrote the manuscript. YX, BY, WG, MS, MY, YJ, and HL analyzed, interpreted the data, and performed the clinical assessment. YJ and HL designed the study. All authors reviewed, edited, and approved the final manuscript.

## Funding

This study was supported by National Natural Science Foundation of China (82070003 and 81770002 to HL), Natural Science Foundation of Hunan Province, China (2021JJ30943 to HL), the Science and Technology Program of Changsha, China (kq1901120 to HL), the Fundamental Research Funds for the Central Universities of Central South University (2021zzts0372 to SD), Xiangya Clinical Big Data System Construction Project in Pulmonary Inflammatory Disease of Central South University, and the National Key Clinical Specialty Construction Projects of China [(2012) No. 650].

## Conflict of Interest

The authors declare that the research was conducted in the absence of any commercial or financial relationships that could be construed as a potential conflict of interest.

## Publisher's Note

All claims expressed in this article are solely those of the authors and do not necessarily represent those of their affiliated organizations, or those of the publisher, the editors and the reviewers. Any product that may be evaluated in this article, or claim that may be made by its manufacturer, is not guaranteed or endorsed by the publisher.
