# Supplementary material for: Case Report: Community-Acquired Legionella gormanii Pneumonia in an Immunocompetent Patient Detected by Metagenomic Next-Generation Sequencing
Source: Front Med (Lausanne). 2022 Jan 28;9:819425. doi: 10.3389/fmed.2022.819425 (PMC8831763; doi:10.3389/fmed.2022.819425)
Supplement: Supplementary file 1 [file Table_1.DOCX]

**Supplementary file: Methods of mNGS**

The overall precedence of mNGS in Changsha Kingmed Medical Test Center Co.Ltd. was similar to that described in a previous published mNGS article which was conducted in Guangzhou Kingmed Medical Test Center Co.Ltd.[1].

**1. Sample Processing**

Bronchoalveolar lavage fluid (BALF) was collected and transported with dry ice. Blood was stored in EDTA tubes, from which plasma was separated by centrifuging at 1600 g for 10 min at 4℃. Metagenomic next-generation sequencing pipeline was conducted in Changsha Kingmed Medical Test Center Co.Ltd..

**2. Sequencing and Quality Control**

In this study, nucleic acid (DNA and RNA) extraction and library preparation were performed on samples through the lab's self-built process. Both nucleic acid extraction and library preparation were conducted in parallel with quality control samples. Qubit was used to measure the concentration of the library. Single-end 75bp sequencing was carried out using Illumina nextseq 550 system with 75 cycles Reagent Kit. Then, the low-quality sequencing data were filtered out. After the removal of the sequences mapped to human reference genome, the remaining data were aligned to the microbial genome database.

**3. Bioinformatic Analysis**

The adapter sequences, low-quality data, and polyG tails were remove to generate the clean data using fastp v0.20.0 [2]. Then sequences that can be mapped to human reference genome were filtered using bwa v0.7.10-r789 [3]. The alignment of the remaining microbial data was carried out using bwa v0.7.10-r789 [3] and Sequence-Based Ultra-Rapid Pathogen Identification (SURPI v1.0.18) pipeline (UCSF), a previously published research pipeline for pathogen identification [4, 5]. For clinical usage, a self-built microorganisms database “MetagenomicX” was used to aligned the sequencing data. The database contains 36497 microorganisms’ genomes, which covers most of the microorganisms which have been sequenced. 8704 of the total microorganisms in the first-grade database have integral sequence of whole genome and detailed clinical analysis, which covers most of the known pathogenic bacteria, viruses, fungi, and parasites. This first-grade database with high-quality genomes is used as first choice.

**References:**

[1]. Shi, Y., et al., A case of chlamydia psittaci caused severe pneumonia and meningitis diagnosed by metagenome next-generation sequencing and clinical analysis: a case report and literature review. BMC Infectious Diseases, 2021. 21(1).

[2]. Chen, S., et al., fastp: an ultra-fast all-in-one FASTQ preprocessor. Bioinformatics, 2018. 34(17): p. i884-i890.

[3]. Li, H. and R. Durbin, Fast and accurate short read alignment with Burrows-Wheeler transform. Bioinformatics, 2009. 25(14): p. 1754-1760.

[4]. Miller, S., et al., Laboratory validation of a clinical metagenomic sequencing assay for pathogen detection in cerebrospinal fluid. Genome Research, 2019. 29(5): p. 831-842.

[5]. Naccache, S.N., et al., A cloud-compatible bioinformatics pipeline for ultrarapid pathogen identification from next-generation sequencing of clinical samples. Genome Research, 2014. 24(7): p. 1180-1192.
